# Sphingolipid Profiling: A Promising Tool for Stratifying the Metabolic Syndrome-Associated Risk

**DOI:** 10.3389/fcvm.2021.785124

**Published:** 2022-01-14

**Authors:** Loni Berkowitz, Fernanda Cabrera-Reyes, Cristian Salazar, Carol D. Ryff, Christopher Coe, Attilio Rigotti

**Affiliations:** ^1^Department of Nutrition, Diabetes and Metabolism & Center of Molecular Nutrition and Chronic Diseases, School of Medicine, Pontificia Universidad Católica de Chile, Santiago, Chile; ^2^Department of Gastroenterology, School of Medicine, Pontificia Universidad Católica de Chile, Santiago, Chile; ^3^Institute on Aging, University of Wisconsin-Madison, Madison, WI, United States

**Keywords:** sphingolipids, cardiovascular risk (CVD), ceramides, metabolic syndrome, type 2 diabetes

## Abstract

Metabolic syndrome (MetS) is a multicomponent risk condition that reflects the clustering of individual cardiometabolic risk factors related to abdominal obesity and insulin resistance. MetS increases the risk for cardiovascular diseases (CVD) and type 2 diabetes mellitus (T2DM). However, there still is not total clinical consensus about the definition of MetS, and its pathophysiology seems to be heterogeneous. Moreover, it remains unclear whether MetS is a single syndrome or a set of diverse clinical conditions conferring different metabolic and cardiovascular risks. Indeed, traditional biomarkers alone do not explain well such heterogeneity or the risk of associated diseases. There is thus a need to identify additional biomarkers that may contribute to a better understanding of MetS, along with more accurate prognosis of its various chronic disease risks. To fulfill this need, omics technologies may offer new insights into associations between sphingolipids and cardiometabolic diseases. Particularly, ceramides –the most widely studied sphingolipid class– have been shown to play a causative role in both T2DM and CVD. However, the involvement of simple glycosphingolipids remains controversial. This review focuses on the current understanding of MetS heterogeneity and discuss recent findings to address how sphingolipid profiling can be applied to better characterize MetS-associated risks.

## Introduction

Cardiovascular diseases (CVD) are currently a major cause of morbidity and mortality with major overall economic healthcare burden worldwide (1). Among these conditions, CVD of atherosclerotic origin (ASCVD) stands out because of its high prevalence and the significant acute ischemic complications and chronic consequences. Many risk factors for ASCVD are well-established, including family history, obesity, blood hypertension, dyslipidemia, type 2 diabetes (T2DM), and proinflammatory pathophysiology (2). In particular, metabolic syndrome (MetS) –a well-known clustering of risk factors (3)– doubles the risk for CVD and increases five-fold the chance for T2DM (4). Indeed, 13.3 to 44% of excess CVD mortality in the US is explained by MetS or MetS-related CVD (5). Furthermore, MetS is associated with increased risk of a number of common cancers (6) and neurodegenerative disorders (7) and is also an important risk factor influencing progression and prognosis of COVID-19 (8).

Based on NHANES reports, the overall prevalence of MetS in US adults was 34.7% in 2016, increasing with age and reaching 48.6% among those aged at least 60 years. Remarkably, over 2011–2016, MetS prevalence increased significantly among those aged 20 to 39 years from 16.2 to 21.3% (9). Thus, prevention, early diagnosis, and appropriate risk stratification of MetS constitute a major health priority challenge. However, besides the many components and clinical implications of MetS, there is no agreement upon clinical definition of MetS. Moreover, the pathophysiology of MetS is not consensual (10), and traditional biomarkers alone do not explain its heterogeneity or the specific risk of associated chronic diseases.

In search for new biomarkers, and in line with the refinement of new omics technologies, several studies have focused on bioactive sphingolipids (SPLs) (11). SPLs have emerged as signaling molecules that regulate many metabolic functions, and ample evidence highlights their role in the regulation of inflammatory responses (12). A substantial body of literature shows that ceramides, a major key class of this lipid family, may have a causative role in diabetes and ASCVD (13). However, the involvement of more simple glycosphingolipids (e.g., hexosylceramides and lactosylceramides) is still controversial.

This review focuses on the current understanding of MetS heterogeneity as well as recent findings that address how sphingolipid profiling may provide additional and valuable information to better characterize MetS-associated risk.

### Emerging Cardiovascular Risk Factors, Including Sphingolipid Biomarkers

There are now many clinical guidelines for evaluation and management of ASCVD risk. The most common factors included in current risk calculators are age, sex, total cholesterol and HDL cholesterol (HDL-c) levels, blood pressure, diabetes, and smoking (14). Some guidelines also add in LDL cholesterol (LDL-c) levels, obesity, and high sensitivity C-reactive protein (15). However, there continue to be deficiencies in modeling risk as well as differences in the impact of each component (14). Indeed, estimations indicate that traditional risk algorithms may miss up to 20% of future CVD events (16).

The pursuit of better strategies for risk prognosis is under continuous evolution. For instance, although plasma LDL-c levels have been considered a primary etiopathogenic factor for development of ASCVD, currently the concentration of non-HDL-c is considered a better predictor of future CVD events (14, 17). Similarly, apolipoprotein B (Apo B) –the structural protein present in all atherogenic lipoproteins– and lipoprotein(a) have also been proposed as emerging cardiovascular risk factors. On the other hand, the recognition of ASCVD as an active process of vascular damage –rather than passive cholesterol infiltration of blood vessels– has highlighted inflammatory and procoagulant mechanisms and biomarkers. In this context, several proinflammatory and prothrombotic molecules (i.e., C-reactive protein, fibrinogen, IL-6, sICAM, etc.) are being assessed to add predictive power to existing CVD risk models (18, 19).

Currently, some of the most promising CVD risk prediction approaches are panels of multiple circulating biomarkers. In fact, omics technologies have provided new insights into the association between a wide variety of novel plasma molecules and cardiometabolic disorders (20). Particularly, based on the technological advances in lipidomics, researchers have identified nontraditional and less abundant lipids as possible biomarkers of early stage cardiometabolic dysfunction as well as cardiometabolic risk (21, 22). Indeed, multiple studies have identified non-traditional lipid species or lipidomic profiles related to subclinical atherosclerosis (23), future cardiovascular events (24–27), and CVD mortality (28). Most of them improved prediction of CVD beyond the sensitivity of traditional cardiovascular risk factors.

Moreover, longitudinal lipidomic phenotypes seem better predictor of future risk of ASCVD in healthy adults. One study showed that numerous non-cholesterol lipids, especially sphingolipid, phospholipid, diacylglycerol, and triglyceride species, deserved more consideration in CVD risk stratification in patients with low-risk cholesterol profiles (29). Similarly, two additional classes of circulating lipids (e.g., dihydroceramides and lysophosphatydilinositol) may serve as novel biomarkers to identify individuals at high-risk of diabetes prior to disease onset (30). In this context, a substantial body of evidence shows that some species of ceramides, a key class of the sphingolipid family, may play a causative role and be relevant for risk prediction of various cardiometabolic disorders, including CVD and diabetes (31–33). These observations have increased the pathophysiological interest and diagnostic potential of sphingolipids in MetS, a condition where the risk of CVD and diabetes converge, but with a highly heterogeneous pathobiology.

### MetS Controversies in Definition and Chronic Disease Risk Association

MetS is a multicomponent condition that reflects the clustering of individual cardiometabolic risk factors related to abdominal obesity and insulin resistance. There are various definitions–based on shared elements–that somewhat have been agreed upon by different international organizations and expert groups.

A number of the most commonly used definitions are those proposed by the World Health Organization (WHO), the European Group for the Study of Insulin Resistance (EGIR), the National Cholesterol Education Program Adult Treatment Panel III (NCEP:ATPIII), the American Association of Clinical Endocrinology (AACE) and the International Diabetes Federation (IDF) (34). Most organizations recommend a harmonized definition for MetS based on the presence of any 3 of the following 5 risk factors: abdominal obesity, low HDL-c levels, high triglycerides levels, high blood pressure, and hyperglycemia (35). This delineation is congruent with the criteria outlined by the NCEP-ATPIII, one of the most popular definitions of MetS. The IDF definition has the same criteria but considers abdominal obesity as a requirement for MetS diagnosis. Conversely, AACE, WHO, and EGIR definitions regard insulin resistance as central to the pathophysiology of MetS and thus must be present for diagnosis (34).

The need to define MetS more precisely stems from the importance of correctly identifying individuals at high risk for ASCVD vs. other chronic diseases. Several epidemiological studies have confirmed the increased risk of ASCVD in individuals with MetS, regardless of the diagnostic criteria used (36–38). However, there continues to be an ongoing controversy about whether MetS is in fact a homogeneous clinical condition, disorder, or disease (39), and whether it requires recognition as a specific syndrome. Moreover, the predictive value of MetS has been questioned because its detection may not provide additional information than its individual components (40). On the other hand, even though MetS has been associated with higher odds of cardiovascular events and T2DM, it remains unclear whether MetS is a single syndrome or a constellation of different attributes or traits conveying a divergent range of metabolic vs. cardiovascular risk (39). Using a continuous MetS severity scale, a study confirmed that a more severe presentation of this syndrome quadruples the risk of coronary events (41). But there are likely to be differences in the associated risk depending on the presence of specific MetS components as well other factors that remain to be defined. For instance, the combination of central obesity, elevated blood pressure, and hyperglycemia conferred the greatest risk for CVD and mortality in the Framingham Offspring Study (42). However, there is a lack of biomarkers that allow a more accurate prediction of a patient's risk progressing to one of the various pathologies associated with MetS (i.e., T2DM, CVD, Alzheimer's disease, cancer).

Traditional serum lipid biomarkers of cardiovascular health include low triglycerides as well as high HDL-c and low LDL-c (2). However, patients with MetS show several lipid abnormalities beyond high LDL-c, elevated triglycerides, or low HDL-c, such as high levels of modified LDL particles (43). In fact, traditional lipid measures alone do not fully explain the complexity of the altered lipid metabolism associated with MetS or its related high cardiovascular risk (CVR) (43). Moreover, currently used risk prediction calculators and available therapies are insufficient and a significant amount of undefined residual CVR remains undetected and untreated (44). In this context, biomarkers beyond those included in the existing definitions of MetS, such as endothelial dysfunction, prothrombotic tendency, and proinflammatory state, may be essential determinants of future CVR in MetS patients (45). However, these risk biomarkers would not provide information regarding the underlying metabolic status, and the risk of other pathologies such as T2DM. As described below, profiling of circulating sphingolipids could contribute to a better pathophysiological understanding of MetS, with more accurate prognosis of cardiovascular vs. diabetic risks.

### Sphingolipids

Sphingolipids (SPL) are a highly diverse group of biomolecules that are not just structural components of cell membranes, but also participate in intra- and extracellular signaling. SPL are found in a wide variety of organisms and are as a matter of fact involved in many aspects of cell structure, recognition, metabolism, and regulation (46).

**Figure 1:**
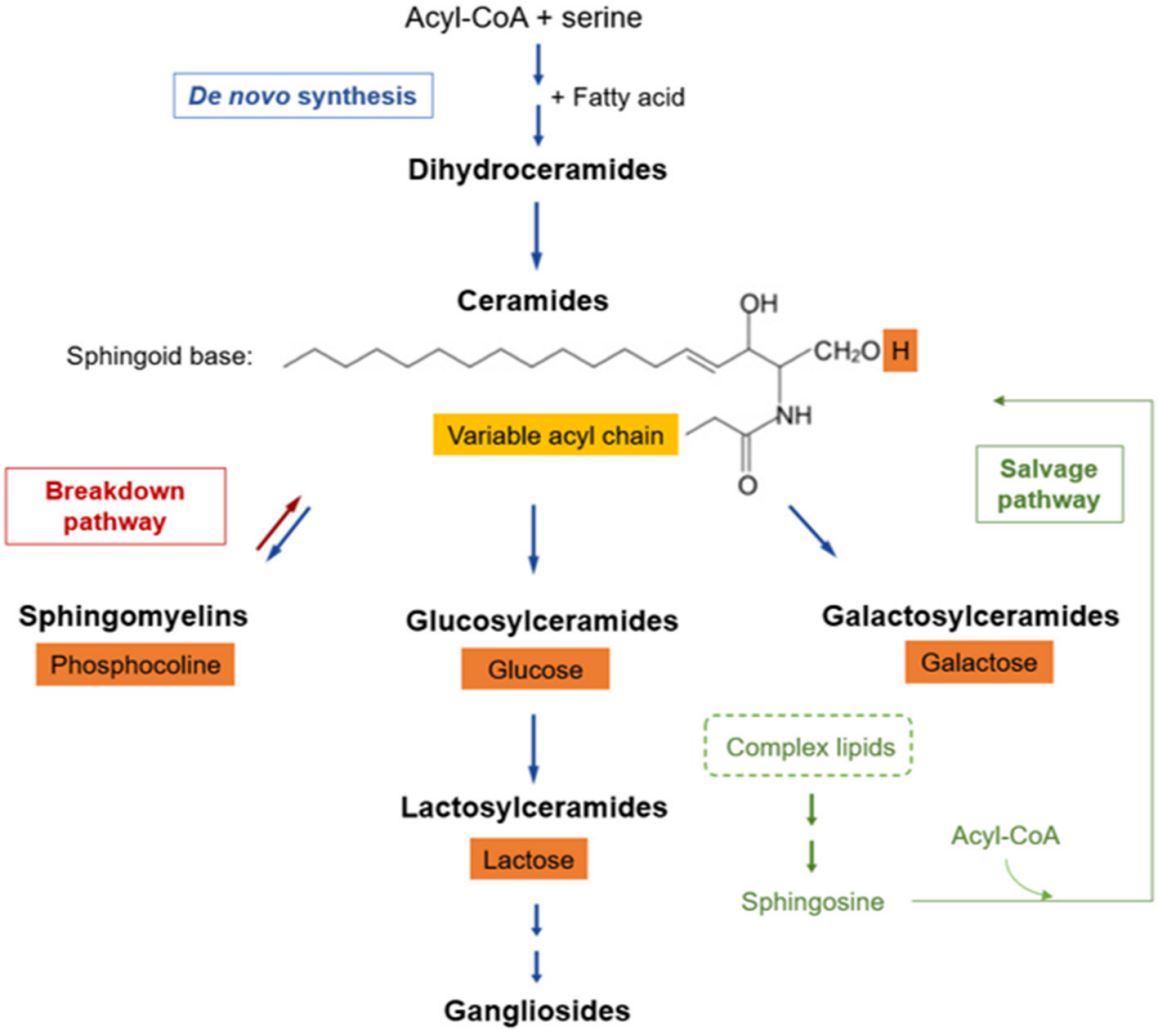
Sphingolipid metabolic pathway. SPLs have three major metabolic pathways, all of which converge into ceramides: (a) *de novo* synthesis coming from saturated fatty acids, (b) breakdown pathway in which sphingomyelin loses its phosphocholine headgroup, and (c) salvage pathway that allows sphingosine recycling from complex SPLs. The fatty acid (light orange box) defines SPL species, whereas the headgroup (dark orange boxes) defines the SPL class.

SPL comprise a complex family of compounds structurally defined by a backbone called “sphingoid” base, mostly represented by sphingosine, which is amide-linked with long- or very-long-chain fatty acids to form ceramides (Figure 1). Ceramides can be further derivatized –by addition of a headgroup– to form more complex SPL classes, such as sphingomyelin, simple glycosphingolipids (e.g., glucosylceramides, galactosylceramides and lactosylceramides), and more complex glycosphingolipids with a few to dozens of sugar residues, called gangliosides (46). Within each SPL class, there are many species components defined by structural and chemical features (i.e., carbon chain length, double bonds) of the attached fatty acid.

At a cellular level, SPL are found mainly in the plasma membrane, where they interact with cholesterol forming membrane microdomains known as “lipid rafts”, which regulate signal transduction and cell recognition. In circulation, >500 species of plasma SPLs are transported in the different classes of lipoproteins (i.e., VLDL, LDL, HDL) or associated with albumin (47, 48). Lipoprotein-associated SPL can be incorporated into these lipid particles before they are secreted from cells (49), transferred from one lipoprotein to another, or modified within them (47). Consequently, changes in circulating lipoprotein levels may affect circulating sphingolipid levels (48, 50–53).

### SPL Profile and Metabolic Complexity

SPL metabolism can be thought of as a discrete set of three metabolically connected pathways: (a) *de novo* synthesis coming from saturated fatty acids, (b) salvage of sphingosine, and (c) breakdown of complex SPLs (46), all of which converge into ceramides (Figure 1). In *de novo* synthesis, an acyl-CoA condensates with serine, which –by addition of another fatty acid– subsequently gives rise to dihydrosphingosine. Then, sequential enzymatic reactions lead to the generation of dihydroceramide, the reduced precursor of ceramides. Some dihydroceramides are not transformed further and they *per se* participate in signaling pathways while another fraction is oxidized into ceramides (46, 54). Afterwards, ceramides can be modified by incorporation of a polar group, such as phosphocholine, originating sphingomyelin, or sugars, giving rise to a wide range of glycosphingolipids (a.k.a. glycoceramides).

Many glycosyltransferases are involved in the synthesis of glycosphingolipids, starting with the addition of glucose or galactose, leading to the so-called hexosylceramides. Within these, glucosylceramide is the major form, and it may be subsequently modified by lactosylceramide synthase, which adds galactose giving rise to lactosylceramide (46, 55). Hexosylceramides and lactosylceramides are simple β-glycosphingolipids that can be further glycosylated, generating more complex glycosphingolipids (e.g., gangliosides), or remain as such leading to their biological activities (56).

SPL metabolism is interconnected with pathways involved in oxygen biology, immune response, glycolysis, amino acid metabolism, and metabolism of other lipids (46). Hence, there are multiple factors that potentially modify the SPL profile. For example, increases in reactive oxygen species (57) or a hypoxic state (58) predispose to accumulation of dihydroceramides. On the other hand, high levels of circulating saturated fatty acids stimulate a dose-dependent synthesis of ceramides (13, 59), which accounts for the accumulation of ceramides in overnutrition or dyslipidemia (50). Similarly, proinflammatory cytokines, such as TNF-α, can stimulate the hydrolysis of sphingomyelin to ceramide (60), while bacterial antigens (e.g., LPS) promote the synthesis of some glycosphingolipids (61). Both LPS and saturated fatty acids also activate the toll-like receptor 4 (TLR4) and induce the transcription of genes associated with SPL synthesis (61). More distant modulating factors, such as lower psychological well-being, which is linked with an unhealthy lifestyle, has also been associated with higher levels of ceramides (62). Taken together, these findings show that SPL circulate in a dynamic balance that is likely to be altered under various physiological and disease conditions.

### Biological Functions of SPLs Related With Cardiometabolic Diseases

Bioactive lipids are functionally defined as those lipid species whose levels respond to the action of specific stimuli and then regulate distinctive downstream effectors and targets. The most studied bioactive SPLs are ceramides. However, several additional SPL species are being evaluated as potential bioactive lipids (55). Thus, a major challenge is to keep up with the rapid growth in knowledge about the sphingolipidome (i.e., the ensemble of all SPL in living organisms) as well as its physiological and pathogenic impact.

The range of functions currently attributed to bioactive SPLs is wide and includes many aspects of cell biology and body physiology, such as cell growth and cell cycle, cell senescence and death, inflammation, immune response, cell adhesion and migration, angiogenesis, metabolism, and autophagy (55). Considering this wide variety of functions, SPLs have been implicated in several pathological disorders, including obesity, T2DM, atherosclerosis and CVD (11, 63, 64) (Table 1). However, many of the underlying mechanisms involved remain unclear and vary depending on the specific class, or even species, of SPL. For instance, ceramides elicit metabolic dysfunction by several mechanisms. They inhibit protein kinase B (Akt/PKB) via protein phosphatase 2 (PP2A) and protein kinase C zeta (PKCz) intermediaries, which mediate a wide range of downstream effects, including insulin resistance (55, 67, 68, 93). In addition, ceramide acts as a second messenger in activating the apoptotic cascade in many cell types, including β cells in pancreatic islets (33, 94). Ceramide overload also induces endoplasmic reticulum stress, inhibits mitochondrial fatty acid β-oxidation, and activates the NLRP3 inflammasome (13), pathophysiological processes associated with obesity.

**Table 1:** Association between sphingolipid classes and cardiometabolic disorders.

**Sphingolipid class**	**Relationship with**	**Proposed mechanisms**	**Most studied SPL species**	**Supporting evidence**
	**Obesity**			
Ceramides	↑	Visceral obesity promotes the SPL biosynthetic pathway, increasing circulating ceramides.	Cer-C16 and Cer-C18	*In vitro* and animal models (61, 65, 66), cross-sectional studies (21)
HexCer/LacCer	↓ or =	Unclear. Upregulation of salvage pathway	Sphingosine-based HexCer and Hex2Cer species	*In vitro* (49), cross-sectional studies (21)
	**Dyslipidemia**			
Ceramides	↑c-LDL, ↑TGs, ↓c-HDL	Bidirectional. Higher levels of ApoB-containing lipoproteins increase ceramide circulation. Ceramide depletion could accelerate the catabolism of ApoB-containing lipoproteins.	-	*In vitro* (48), animal models (50), cross-sectional studies (21, 51), prospective studies (52)
HexCer/LacCer	↓c-LDL, ↓TGs, ↑c-HDL	Unclear. Upregulation of salvage pathway		*In vitro* (53), cross-sectional studies (21)
	**Dysglycemia**			
Ceramides	↑	Ceramide accumulation would alter glucose metabolism, by inhibition of Akt/PKB, inducing insulin resistance, and by stimulation of β cells apoptosis in pancreatic islets, increasing the risk of diabetes.	Cer-C18 and C20	*In vitro* (67–69), animal models (70–72), cross-sectional studies (73), prospective studies (21, 64)
HexCer/LacCer	↓	Increased synthesis of hexosylceramides at expense of ceramides would enhance insulin sensitivity by ceramide reduction, and by immunomodulatory actions.		*In vitro* (69), animal models (74, 75), cross-sectional studies (21), prospective studies (74–76)
GM3	↑	GM3 would cause insulin resistance, by reduction of insulin receptor presentation on fat cell surface due to changes in composition of lipid rafts.		*In vitro* and *in vivo* (77)
	**Cardiovascular disease**			
Ceramides	↑	Ceramides increase CV risk, by increasing transport, retention, and aggregation of ceramide-enriched LDL within the vascular wall; apoptosis of cells lining the vascular wall, and reduction of vasorelaxation and fibrinolysis.	Cer-C16, C18 and C24: 1 and their ratio over Cer-C24	*In vitro* (14, 78), animal models (79, 80), and prospective studies (81–84)
HexCer	↑	GluCer regulate downstream signaling of LPS/TLR4, increasing secretion of proinflammatory cytokines.		*In vitro* (85, 86)
LacCer	↑	LacCer increase CV risk: lead to oxidative stress environment and upregulate adhesion molecules on vascular endothelial cells and monocytes.		*In vitro* (87–90), animal models (91), prospective studies (22, 24)
GM3	↑	GM3 would increase foam cell formation		*In vitro* (92)

*The arrows symbolize the relationships between blood SPL levels and MetS-associated conditions. An upward arrow indicates a positive association and a downward arrow indicates a negative association*.

On the other hand, glycosphingolipids fulfill most of their functions by structuring glycosphingolipid-enriched microdomains in cell membranes. Of particular importance is the assembly of these glycosphingolipids with signal transducers and other membrane proteins to form functional signaling units (95). For example, studies showed that GM3, a complex ganglioside, reduced insulin receptor presentation on the cell surface of adipocytes by modifying the composition of lipid rafts (77). However, this alteration in insulin signaling does not occur with simpler glycosphingolipids or in other types of cells, where the effect could even be favorable (69).

In the same line, glycosphingolipids are important determinants of a functional immune system. Glycosphingolipid repertoires present in the plasma membrane of immune cells impact membrane organization, molecular signaling, cell differentiation, and trans-interaction between biomolecules located in adjacent cell surfaces (96). Glycosphingolipid-enriched plasma membrane microdomains mediate immunological and inflammatory reactions, including superoxide generation, chemotaxis, and non-opsonic phagocytosis (97). In addition, lactosylceramides activate cytosolic phospholipase A2 (cPLA2), which cleaves arachidonic acid from phosphatidylcholine to generate eicosanoids and prostaglandins, two essential inflammatory intermediaries (87, 98). As detailed below, the role of glycosphingolipids in inflammation and immune response has been directly related to the development and progression of ASCVD.

All these findings have increased interest in verifying the usefulness of measuring plasma SPL levels as new biomarkers. However, these analyses have been hampered by the complexity and diverse functions of this lipid family. Therefore, it remains a significant need to better define sphingolipidome profiles under normal and pathological conditions to further understand how absolute levels of different SPLs and, eventually, the relative ratio between them regulate cell function/body physiology and are associated with the origin and progression of chronic diseases. In fact, we must establish more precisely the relevance of SPL profiles to risk stratification and prognosis of MetS, where all the metabolic disorders mentioned above converge.

### SPLs as Potential Mediators of Inflammation in Cardiometabolic Diseases

#### Studies *in vitro*, Cultured Cells, and Animal Models

Given their role in metabolic and inflammatory processes, there is an emerging interest in elucidating the association of SPLs and ASCVD. Ceramides have been shown to be relevant for understanding the pathophysiology of the atherothrombotic process and exert several specific actions during plaque formation and ischemic complications (79, 80) (Table 1). For example, generation of ceramide by sphingomyelin-breakdown induction was found to be sufficient in activating the aggregation of lipoproteins *in vitro* (99, 100). In fact, aggregated LDL particles obtained from atherosclerotic lesions have 10- to 50-fold higher levels of ceramides than plasma LDL particles (101). Ceramide was also implicated in transcytosis of oxidized LDL across endothelial cells, thus leading to the transport and retention of atherogenic lipoproteins within the vascular wall (102). Likewise, the acute induction of ceramide synthesis by monocytes increased adhesion of these proatherogenic lipoproteins to rat aortic endothelium and uptake of oxidized LDL *in vitro* (103). Moreover, ceramides have been shown to induce apoptosis of cells lining the vascular wall, a process implicated in plaque rupture during acute ischemic complications of atherothrombotic disease (78). Finally, ceramides impair endothelium-dependent vasorelaxation and fibrinolysis *in vitro* (13) further increasing the risk for atherothrombotic events. In parallel, several studies using cell and animal models have also shown that ceramides impair glucose metabolism in different cell types and organs, including pancreas, skeletal muscle, and adipose tissue (33, 70–72, 104, 105), increasing T2DM risk (64)–a prime risk factor for ASCVD.

In contrast to unglycosylated ceramides, the involvement of glycosphingolipids in cardiometabolic diseases is more controversial. Specifically, simple –rather than complex– glycosphingolipids seem to have a protective role against metabolic disorders (Table 1). For example, administration of simple β-glycosphingolipids showed remarkable beneficial effects on glucose intolerance and hepatic steatosis in mice (74), rats (75), and *Psammomys obesus* (76). This effect was attributed to the immunomodulatory role of this β-glycosphingolipid on immune cells involved in these disorders (74–76). As well, induction of glucosylceramide synthesis in myotubes enhanced insulin signaling (69).

In contrast, elevated levels of lactosylceramide –which were accompanied with decreased respiration and calcium retention capacity in mitochondria– have been reported in heart tissue of streptozotocin-induced type 1 diabetes mouse model (106). These metabolic changes may impair immune cell function (107). In addition, lactosylceramides lead to oxidative stress environment in human aortic smooth muscle cells by activating NADPH oxidase and generating reactive oxygen species (108). Therefore, despite the beneficial effects that these glycosphingolipids may have on glucose metabolism, lactosylceramides would contribute to mitochondrial dysfunction, oxidative stress, and inflammatory response in diabetes.

In addition, the involvement of lactosylceramides and hexosylceramides on ASCVD has been reported in several models (Table 1). For example, atherogenic apoE knockout mice exhibit increased serum concentrations of glycosphingolipids and accumulation of specific glycosphingolipids in atherosclerosis-prone regions of the aorta (91, 109). In keeping with this finding, using cell lines and mouse models, several immune mechanisms–as follows–have been proposed to account for a direct proatherogenic effect of specific glycosphingolipids. A recent study reported that macrophages accumulated ceramides and glucosylceramide in response to inflammatory activation with IFN-γ and LPS (110). Interestingly, glucosylceramide present in plasma membrane microdomains regulate LPS/TLR4 orientation, affecting downstream signaling proteins of this complex and the production of proinflammatory cytokines, as IL-6 and TNF-α (85, 86). On the other hand, lactosylceramide induced smooth muscle cell proliferation *in vitro* (88) while ganglioside GM3 markedly accelerated LDL uptake by macrophages, leading to generation of lipid-laden foam cells (92). On the other hand, *in vitro* studies, evaluating the pro-inflammatory role of β-glycosphingolipids, suggested that lactosylceramides may upregulate adhesion molecules on vascular endothelial cells and monocytes as well as activate neutrophils, contributing to atheromatous plaque inflammation (87, 89, 90). Moreover, a murine model showed that lactosylceramide is enriched in plasma membrane microdomains of neutrophils and involved in cell migration and phagocytosis (97). Indeed, inhibition of glycosphingolipid synthesis helped to preserve cardiac function in an animal model of diet-induced ASCVD (111).

On the other hand, peripheral monocytes isolated from healthy humans exhibited enhanced migration when incubated with exogenous lactosylceramide (112). Specifically, lactosylceramides increased the expression of ICAM-1 in human endothelial cells, through NADPH oxidase activation and ROS generation (113). Similarly, lactosylceramides induced the expression of CD11/CD18 in human neutrophils and monocytes, facilitating their adhesion to the endothelium and entry into the intimal space (87, 98). All these lactosylceramide-associated processes would contribute to inflammation and development of atherosclerosis. Finally, glycosphingolipids can also modulate the adaptative immune response in ASCVD. IFN-α, a proinflammatory cytokine found in atherosclerotic plaques, upregulated lactosylceramide and glucosylceramide production in mouse B cells (114). These lipid classes are a fundamental component of glycosphingolipid-enriched microdomains involved in activation and proliferation of B and T lymphocytes, which modulate and regulate amplification of an inflammatory response (96).

### Human Clinical and Epidemiological Studies

More recently, several studies in humans have shown an association between ceramides and cardiometabolic diseases. High levels of plasma ceramides were detected in patients with T2DM (33), arterial hypertension (115), and atherosclerosis (116). Furthermore, accumulation of ceramides in atheromatous plaques seems to stimulate apoptosis of vascular cells, destabilizing the plaque, and thus favoring acute ischemic events (14, 78). In agreement with this perspective, multiple studies have confirmed the association between ceramides and cardiovascular events, even after adjusting for all other well-known risk factors (81–84).

Among the ceramide species studied in humans, whereas Cer-C18 and C20 have been the most associated with T2DM, Cer-C16, C18 and C24:1 as well as their ratio over Cer-C24 have been the species mainly correlated with CVD (11, 73) (Table 1). Based on this type of evidence, a blood ceramide-based diagnostic CVR test (Ceramide Risk Score) was commercialized in 2016 (117). However, one limitation when interpreting the results from this test is that ceramides may be raised in response to different inflammatory states, which are not necessarily indicative of ASCVD (117). Moreover, basal SPL levels also evince considerable variation across different racial and ethnic populations (118). Therefore, more basic and clinical research is still required to further characterize SPL profiles before they can be employed as a diagnostic test with high sensitivity and specificity in routine medical practice.

As mentioned above, the relationship between glycosphingolipids and abnormal cardiometabolic processes remain controversial, but as this article explains, it seems dependent on the pathophysiological context. For example, three recent studies–based on different methodologies and populations–found a negative correlation between hexosylceramide levels and clinical and biochemical features of obesity and diabetes (21, 119, 120). Furthermore, a negative association between simple β-glycosphingolipids and the diagnosis of T2DM was found during follow-up (21). Interestingly, these observations have not been limited to only a few molecular forms, but to multiple species of hexosylceramides and lactosylceramides (21, 119, 120). In sum, emerging evidence seems to indicate that low circulating levels of simple β-glycosphingolipids and high levels of ceramides (i.e., high ceramide/simple β-glycosphingolipids ratio) would be consistently associated with glucometabolic disorders.

Conversely, in the last years, two large prospective cohort-based studies of lipidomic profiles positively associated both ceramides and simple β-glycosphingolipids with future cardiovascular events and cardiovascular death, even in patients with T2DM and after adjusting for traditional risk factors (22, 24). Similarly, levels of lactosylceramides, glucosylceramides, and dihydroceramides were directly correlated with levels of macrophages, IL-6, and macrophage inflammatory protein-1β in human carotid plaques (89). Moreover, a strong link between pulse wave velocity and arterial stiffness, as preclinical atherosclerotic biomarkers, with plasma lactosylceramide levels was found in overweight middle-aged subjects who had fasting hyperglycemia (121). Therefore, despite the relationship with better glucose metabolism, all these studies suggest a potential pro-atherogenic role of simple β-glycosphingolipids, consistent with the aforementioned *in vitro* studies.

### SPLs and MetS Associated-Risk Stratification

Several lipidomic studies have in fact identified novel biomarkers linked to metabolic syndrome or its components (122–124). However, most of them are cross-sectional studies or consider MetS as a single disorder. To our knowledge, only one study evaluated lipid species associated with longitudinal changes in MetS components (125). Whereas lysophosphatidylcholine species were correlated with lower BMI and glycemia, two sphingomyelin species were associated with an increased blood glucose levels during follow-up. However, it remains unclear if these lipid signatures could indeed predict the future risk of diseases associated to MetS.

**Figure 2:**
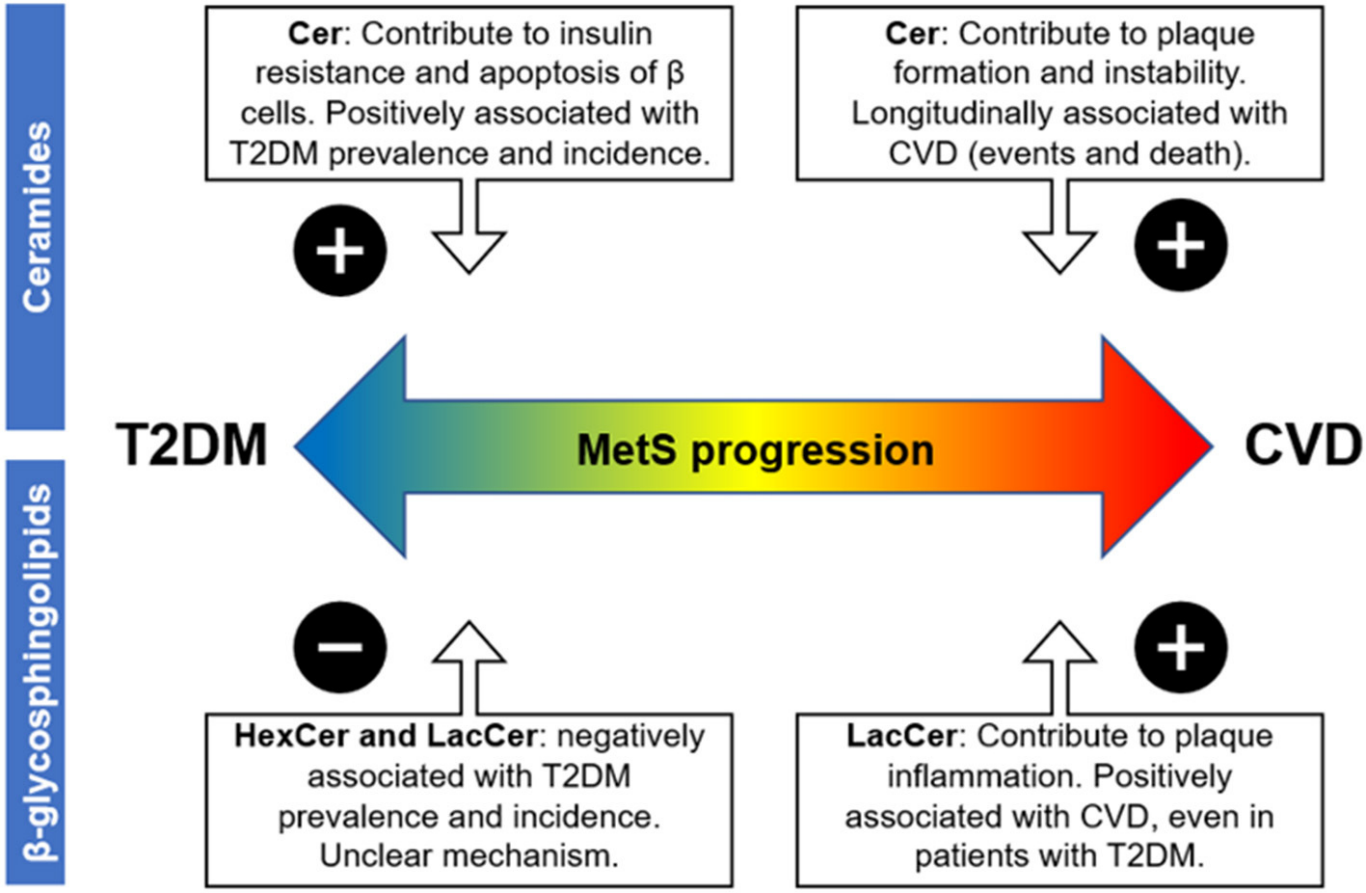
Possible impact of blood sphingolipid profile on MetS-associated cardiovascular vs. metabolic risk based on cross-sectional and longitudinal association studies. Abbreviations are as follows: metabolic syndrome (MetS), atherosclerotic cardiovascular disease (ASCVD), and type 2 diabetes mellitus (T2DM). The “plus sign” denotes a positive association between the respective disease progression and the sphingolipid class, while the minus sign denotes a negative association.

As mentioned above, there is a variable, and even divergent, relationship between different sphingolipid classes and cardiometabolic conditions such as dyslipidemia, insulin resistance, obesity, and atherosclerosis (Figure 2). Thus, it seems appropriate to hypothesize that further sphingolipid profile characterization may contribute to a better understanding of a complex clinical condition such as MetS, in which different cardiometabolic alterations converge and progress heterogeneously. However, it should be noted that although various studies relate sphingolipids with specific cardiometabolic alterations, much of its potential as biomarkers is based primarily on cohort association studies. For example, several lipid profiling studies have reported that circulating dihydroceramides were strong prognostic indicators of future glucometabolic dysfunction (126, 127). Even so, dihydroceramides would not be causative, but most likely would serve as markers of increased flux of fatty acids due to insulin resistance through the ceramide biosynthetic pathway (70).

Regarding specific components of the MetS definition, the associations of sphingolipids with obesity and insulin resistance have been well characterized in humans and animal models (13, 65, 66). Overnutrition and visceral obesity promote SPLs synthesis and turnover, which in turn remodels SPLs profiles and their topological distribution within and between cell membranes, thus defining organelle structure and function (33, 54, 125). In this context, ceramide accumulation in tissues may be impairing many metabolic processes that underlie diabetes and diabetic complications, according to the mechanisms already described (13). Interestingly, Chaurasia et al. demonstrated that inhibition of the enzymatic transformation of dihydroceramides to ceramides in obese mice resolved hepatic steatosis and insulin resistance, suggesting that ceramide actions could not be recapitulated by dihydroceramides (70).

On the other hand, hexosylceramides and lactosylceramides have an immunomodulatory role, and their overall effect would depend on the overall pathophysiological context. Regarding metabolic risk, several studies have consistently shown an inverse relationship between plasma levels of simple β-glycosphingolipids and the prevalence and incidence of diabetes, using different methodologies and based on various population cohort (21, 119, 120). Although the mechanism is not clear, administration of these glycosphingolipids improved glucose intolerance in different animal models of diabetes (74, 75), suggesting a causal role. Therefore, in patients with MetS and overweight, high circulating levels of ceramides would increase the risk of diabetes, whereas this risk could be counterbalanced by the concentrations of simple glycosphingolipids.

Conversely, both ceramides and simple β-glycosphingolipids have been positively associated with ASCVD, as mentioned above. Increased levels of ceramides were related to aggregation of LDL particles in atherosclerotic lesions, induction of apoptosis of cells lining the vascular wall, and platelet activation and aggregation (100, 102, 103). On the other hand, lactosylceramides may upregulate adhesion molecules on monocytes and vascular endothelial cells as well as activate neutrophils, contributing to plaque inflammation and instability (98). In fact, human studies show that both ceramides and simple β-glycosphingolipids are associated with the occurrence of cardiovascular events and cardiovascular death, even in patients with T2DM (22). Therefore, despite the beneficial effects that simple β-glycosphingolipids may have on glucose metabolism and intolerance, both ceramides and simple β-glycosphingolipids may contribute to cardiovascular risk in patients with MetS. Interestingly, a recent study showed that statin therapy did not significantly lower circulating concentrations of these two sphingolipids (128). Therefore, high levels of ceramides and glycosphingolipids could account for a fraction of the residual cardiometabolic risk in statin-treated MetS patients.

Based on all this background, SPL profiling may provide novel and relevant insights into the burden of cardio vs. metabolic risk in patients with MetS. Although abdominal obesity seems to increase all classes of sphingolipids, a blood profile characterized by high levels of ceramides and low levels of simple β-glycosphingolipids in MetS patients may implicate a higher risk of T2DM (Figure 2), based on the evidence discussed here. On the other hand, a profile characterized by high levels of both ceramides and simple β-glycosphingolipids during MetS could indicate a more inflammatory and pro-atherogenic state, and therefore, a higher risk of ASCVD (Figure 2).

Importantly, careful consideration of experimental procedures and control variables is required during characterization of sphingolipidomes. Some differences can be observed depending on the analytic technique used, the origin of the sample (e.g., serum vs. plasma), and the feeding condition of the subject (e.g., fasting vs. non fasting) (20, 129). Furthermore, it is also necessary to evaluate the representativeness of these biomarkers among different subpopulations, since it could vary by racial, sex or age groups (130, 131). For example, there are ethnic and racial differences in the prevalence of MetS and its components (132). In general, African-Americans have lower prevalence of MetS when compared to whites, but suffer disproportionately from higher cardiovascular mortality and T2DM (132). Thus, further research is needed to explore the potential applications of SPL profiling to improve MetS-associated risk prediction in this population.

## Conclusion

Stratification and better risk prediction of MetS constitutes a health priority and challenge. Traditional biomarkers alone do not explain its heterogeneity or the specific future risk of associated diseases. Moreover, even though MetS has previously been linked with higher odds of cardiovascular events, little is known about specific clusters of MetS components and their associated-risk differences for development of ASCVD vs. T2DM.

The evidence discussed in this review suggests that sphingolipid profiling appears as a promising tool for MetS-associated risk stratification. Evaluation of simple β-glycosphingolipids, in addition to more commonly assessed ceramide species, may provide relevant insights into the burden of dysmetabolic state vs. inflammatory state in patients with MetS. Based on this information, the sphingolipid profile –as an additional laboratory test– may have the potential to greatly improve the ability to distinguish MetS patients at risk of suffering a cardiovascular event in the short/medium term from those patients more likely to develop diabetes in the future. We are currently carrying out prospective cohort studies that will be critical to evaluate whether different SPL profiles allow a better classification of MetS patients based on their clinical progression.

## Author Contributions

LB and AR: conceptualization. LB, FC-R, CS, and AR: writing—original draft preparation. LB, FC-R, CS, CR, CC, and AR: writing—review and editing. All authors contributed to the article and approved the submitted version.

## Funding

Preparation of this manuscript was supported by the National Fund of Scientific and Technological Development (Postdoctoral FONDECYT Grant #3210391 and regular FONDECYT Grant #1201607) from the Government of Chile and by the National Institute on Aging (Grant #P01 AG020166).

## Conflict of Interest

The authors declare that the research was conducted in the absence of any commercial or financial relationships that could be construed as a potential conflict of interest.

## Publisher's Note

All claims expressed in this article are solely those of the authors and do not necessarily represent those of their affiliated organizations, or those of the publisher, the editors and the reviewers. Any product that may be evaluated in this article, or claim that may be made by its manufacturer, is not guaranteed or endorsed by the publisher.
